# CCR5 ameliorates Japanese encephalitis via dictating the equilibrium of regulatory CD4^+^Foxp3^+^ T and IL-17^+^CD4^+^ Th17 cells

**DOI:** 10.1186/s12974-016-0656-x

**Published:** 2016-07-20

**Authors:** Jin Hyoung Kim, Ajit Mahadev Patil, Jin Young Choi, Seong Bum Kim, Erdenebelig Uyangaa, Ferdaus Mohd Altaf Hossain, Sang-Youel Park, John Hwa Lee, Seong Kug Eo

**Affiliations:** 1 College of Veterinary Medicine and Bio-Safety Research Institute, Chonbuk National University, Iksan, 54596 Republic of Korea; 2 Department of Bioactive Material Sciences, Graduate School, Chonbuk National University, Jeonju, 54896 Republic of Korea

**Keywords:** CCR5, Japanese encephalitis, CD4^+^Foxp3^+^ Tregs, IL-17^+^CD4^+^ Th17, Neuroinflammation

## Abstract

**Background:**

CCR5 is a CC chemokine receptor involved in the migration of effector leukocytes including macrophages, NK, and T cells into inflamed tissues. Also, the role of CCR5 in CD4^+^Foxp3^+^ regulatory T cell (Treg) homing has recently begun to grab attention. Japanese encephalitis (JE) is defined as severe neuroinflammation of the central nervous system (CNS) following infection with mosquito-borne flavivirus JE virus. However, the potential contribution of CCR5 to JE progression via mediating CD4^+^Foxp3^+^ Treg homing has not been investigated.

**Methods:**

Infected wild-type (Ccr5^+/+^) and CCR5-deficient (Ccr5^−/−^) mice were examined daily for mortality and clinical signs, and neuroinflammation in the CNS was evaluated by infiltration of inflammatory leukocytes and cytokine expression. In addition, viral burden, NK- and JEV-specific T cell responses were analyzed. Adoptive transfer of CCR5^+^CD4^+^Foxp3^+^ Tregs was used to evaluate the role of Tregs in JE progression.

**Results:**

CCR5 ablation exacerbated JE without altering viral burden in the extraneural and CNS tissues, as manifested by increased CNS infiltration of Ly-6C^hi^ monocytes and Ly-6G^hi^ granulocytes. Compared to Ccr5^+/+^ mice, Ccr5^−/−^ mice unexpectedly showed increased responses of IFN-γ^+^NK and CD8^+^ T cells in the spleen, but not CD4^+^ T cells. More interestingly, CCR5-ablation resulted in a skewed response to IL-17^+^CD4^+^ Th17 cells and correspondingly reduced CD4^+^Foxp3^+^ Tregs in the spleen and brain, which was closely associated with exacerbated JE. Our results also revealed that adoptive transfer of sorted CCR5^+^CD4^+^Foxp3^+^ Tregs into Ccr5^−/−^ mice could ameliorate JE progression without apparently altering the viral burden and CNS infiltration of IL-17^+^CD4^+^ Th17 cells, myeloid-derived Ly-6C^hi^ monocytes and Ly-6G^hi^ granulocytes. Instead, adoptive transfer of CCR5^+^CD4^+^Foxp3^+^ Tregs into Ccr5^−/−^ mice resulted in increased expression of anti-inflammatory cytokines (IL-10 and TGF-β) in the spleen and brain, and transferred CCR5^+^ Tregs were found to produce IL-10.

**Conclusions:**

CCR5 regulates JE progression via governing timely and appropriate CNS infiltration of CD4^+^Foxp3^+^ Tregs, thereby facilitating host survival. Therefore, this critical and extended role of CCR5 in JE raises possible safety concerns regarding the use of CCR5 antagonists in human immunodeficiency virus (HIV)-infected individuals who inhabit regions in which both HIV and flaviviruses, such as JEV and West Nile virus, are endemic.

## Background

The *Flavivirus* genus, which includes mosquito-borne dengue virus, Japanese encephalitis (JE) virus, and West Nile virus (WNV) [1–3], is associated with significant morbidity and mortality due to fatal hemorrhagic fever and encephalitis. Of the flaviviruses, Japanese encephalitis virus (JEV) continues to be the leading cause of viral encephalitis in Asia and the Western Pacific. It poses an increasing threat to global health and welfare, with approximately 67,900 reported cases annually [4]. Due to rapid changes in climate and demography, JEV is currently spreading to previously unaffected regions such as Indonesia, Pakistan, and northern Australia [5]. The incubation period of JEV ranges from 5 to 15 days and is fatal in 25 to 30 % cases, mostly in infants, and a high proportion of patients who survive have serious neurological and psychiatric sequelae [4], for which JE is considered to be more fatal than WNV encephalitis, resulting in 3–5 % mortality (1100 death/29,000 symptomatic infections) [6]. Pathologically, JE is a severe neuroinflammation in the central nervous system (CNS) closely associated with the disruption of the blood–brain barrier (BBB) [7]. Although little is known about the pathogenesis of JEV, considerable progress has been made in murine models [8, 9]. While JEV infects and kills neurons directly in the CNS, CNS invasion of JEV causes the stimulation of microglia/glia and infiltrated leukocytes, leading to indirect neuronal killing via over-secreting pro-inflammatory cytokines (such as IL-6 and TNF-α) and soluble mediators that can induce neuronal death [10, 11]. This notion implies that JE is an immunopathological disease caused by uncontrolled over-activation of innate and adaptive immune cells, resulting in neurological disorders in the CNS. Therefore, adequate CNS infiltration and activation of peripheral immune cells is considered to play a critical role in protecting hosts from viral encephalitis such as JE. Indeed, CNS infiltration and activation of peripheral leukocytes during JE can cause profound damage if the reaction is excessive or inappropriate [12]. Therefore, balanced CNS infiltration and activation of peripheral leukocytes should be achieved to have a favorable prognosis of JE without tissue injury.

Chemokine-mediated influx of peripheral leukocytes into the CNS is believed to clear infection, but also be responsible for deleterious bystander neuronal damage associated with morbidity and, in some cases, increased mortality. For example, CXCR3-deficient mice are found to have enhanced CNS viral titers and mortality following WNV infection [13], while these mice are protected from lethal infection of lymphocytic choriomeningitis virus (LCMV) or cerebral malaria [14, 15], suggesting that the final outcome of encephalitis will depend on the nature of the pathogen and a range of host factors. Likewise, CCR5 plays a critical role in recovery from flavivirus encephalitis via appropriate CNS migration of peripheral leukocytes, including NK cells and CD4^+^/CD8^+^ T cells [16–18]. Indeed, the important role of CCR5 in human host responses to WNV encephalitis was demonstrated by a retrospective cohort study involving persons homozygous for CCR5Δ32 [19], a loss-of-function mutation found in 1–2 % of Caucasians [20]. Compared to individuals without the mutation, persons carrying a homozygous CCR5Δ32 allele have an increased risk of symptomatic WNV infection. In view of the large number of human infections caused by flaviviruses and their global distribution, there are concerns about the potential adverse outcomes of CCR5 antagonist use for incurable infectious diseases, including human immunodeficiency virus (HIV).

Furthermore, CD4^+^Foxp3^+^ regulatory T cells (Tregs), which regulate excessive immune responses, are preferentially accumulated over effector T cells at sites of disease due to homing signals such as CCR5 [21–24]. CCR5-dependent homing of CD4^+^Foxp3^+^ Tregs at infectious sites in parasitic pathogen infection models has been shown to promote pathogen persistence by regulating the magnitude of pro-inflammatory responses and the equilibrium between IL-17^+^CD4^+^ Th17 and CD4^+^Foxp3^+^ Tregs [23, 24]. Recently, a putative role for CD4^+^Foxp3^+^ Tregs in the pathogenesis of fatal acute inflammatory diseases caused by flaviviruses has been suggested in the context of their regulatory function [25, 26]. However, the role of CD4^+^Foxp3^+^ Tregs in flavivirus encephalitis remains elusive due to a lack of direct evidence. Presumably, CCR5-dependent recruitment of CD4^+^Foxp3^+^ Tregs may affect the progression of viral encephalitis via their regulatory function. To address the direct regulation of JE by CD4^+^Foxp3^+^ Tregs in CCR5-dependent homing context, we examined the role of CCR5 in JE progression using CCR5-deficient (Ccr5^−/−^) mice in this study. Our results revealed that Ccr5^−/−^ mice had exacerbated JE, ultimately resulting in high mortality without altering CNS viral burden, NK response, or T cell response compared to Ccr5^+/+^ mice. However, the increased susceptibility of Ccr5^−/−^ mice to JE was closely associated with decreased ratio of infiltrated CD4^+^Foxp3^+^ Treg to IL-17^+^CD4^+^ Th17 in the CNS. This was directly confirmed by the fact that injection of sorted CCR5^+^CD4^+^Foxp3^+^ Tregs into Ccr5^−/−^ mice provided ameliorated JE without affecting CNS infiltration of IL-17^+^CD4^+^ Th17 cells or inflammatory Ly-6C^hi^ monocytes. Therefore, our data suggest that CCR5 could dictate JE progression by tightly regulating the balance between infiltrated CD4^+^Foxp3^+^ Tregs and IL-17^+^CD4^+^ Th17 cells in the CNS.

## Methods

### Animals

C57BL/6 (H-2^b^) mice (4- to 6-week-old female or male) were purchased from Samtako (O-San, Korea). CCR5 deficient (Ccr5^−/−^) mice and Foxp3^GFP^ knock-in mice (H-2^b^), which co-express EGFP and regulatory T cell-specific transcription factor Foxp3 under the control of an endogenous promoter, were obtained from Jackson Laboratories (Bar Harbor, ME). Ccr5^−/−^·Foxp3^GFP^ mice were generated by crossing Ccr5^−/−^ mice with Foxp3^GFP^ knock-in mice. All mice were genotyped and bred in the animal facilities of Chonbuk National University.

### Cells, viruses, antibodies, and reagents

JEV Beijing-1 strain was obtained from the Green Cross Research Institute (Suwon, Korea) and propagated in a mosquito cell line (C6/36) using DMEM supplemented with 2 % fetal bovine serum (FBS), penicillin (100 U/ml), and streptomycin (100 U/ml) [27]. C6/36 cells were infected with JEV Beijing-1 at a multiplicity of infection (MOI) of 0.1 and incubated in a humidified CO_2_ incubator at 28 °C for 1 h. After absorption, the inoculum was removed and 7 ml of maintenance medium containing 2 % FBS was added. At approximately 6–7 days post-infection (dpi), cultures of host cells showing 80–90 % cytopathic effect (CPE) were harvested. Virus stocks were titrated by conventional plaque assay or focus-forming assay and stored in aliquots at −80 °C until use. Monoclonal antibodies used for flow cytometric analysis and other experiments were obtained from eBioscience (San Diego, CA) or BD Biosciences (San Diego, CA), including fluorescein isothiocynate (FITC)-conjugated anti-CD3ε (154-2C11), Ly6G (1A8), CD8 (53-67), phycoerythrin (PE)-conjugated anti-mouse CD11b (M1/70), Foxp3 (FJK-16s), IFN-γ (XMG1.2), F4/80(BM8), granzyme B (NGZB), peridinin chorophyll protein complex (PerCP)-conjugated anti-mouse Ly6C (HK 1.4), PE-cyanine dye (Cy7)-anti-mouse NK1.1 (PL136), allophycocyanin (APC)-conjugated anti-mouse CD45(30-F11), IL-17 (eBio17B7), TNF-α (MP6-XT22), biotin-conjugated anti-mouse IL-10 (JES5-16E3), and CD49b (DX5). Peptides of the defined I-A^b^-restricted epitopes JEV NS1_132–145_ (TFVVDGPETKECPD), NS3_563–574_ (WCFDGPRTNAIL), and H-2D^b^-restricted epitope JEV NS4B_215–223_ (SAVWNSTTA) were chemically synthesized at Peptron Inc. (Daejeon, Korea). JEV-specific primers for viral RNA detection and primers specific for cytokines, chemokines, and transcription factors (Table 1) were synthesized at Bioneer Corp. (Daejeon, Korea) and used for PCR amplification of target genes.

**Table 1:** Specific primers for the expression of cytokines, chemokines, transcription factor, and JEV RNA used in real-time qRT-PCR

Gene name	Primer sequence (5′-3′)	Position cDNA	Gene bank ID
IL-1β	FP: AAGTGATATTCTCCATGAGCTTTGT	535–559	NM_008361
	RP: TTCTTCTTTGGGTATTGCTTGG	679–700	
IL-6	FP: TGG GAA ATC GTG GAA ATG AG	209–228	NM_031168
	RP: CTC TGA AGG ACT CTG GCT TTG	442–462	
IL-10	FP: CAA CAT ACT GCT AAC CGA CTC CT	253–275	NM_010548
	RP: TGA GGG TCT TCA GCT TCT CAC	405–425	
IL-17	FP: TCT GAT GCT GTT GCT GCT G	87–105	NM_010552.3
	RP: ACG GTT AGA GGT AGT CTG AGG	254–267	
IFN-γ	FP: CAG CAA CAA CAT AAG CGT CA	119–220	NM_008337.3
	RP: CCT CAA ACT TGG CAA TAC TCA		
CCL2	FP: AAA AAC CTG GAT CGG AAC CAA	347–367	NM_011333
	RP: CGG GTC AAC TTC ACA TTC AAA G	426–447	
CCL3	FP: CCA AGT CTT CTC AGC GCC AT	158–177	NM_011337.2
	RP: GAA TCT TCC GGC TGT AGG AGA AG	206–228	
CCL4	FP: TTC TGT GCT CCA GGG TTC TC	128–147	NM_013652.2
	RP: GAG GAG GCC TCT CCT GAA GT	388–407	
CCL5	FP: CCC TCA CCA TCA TCC TCA CT	77–96	NM_013653.3
	RP: CTT CTT CTC TGG GTT GGC AC	275–294	
CXCL1	FP: CGC TGC TGC TGC TGG CCA CC	101–120	NM_008176.3
	RP: GGC TAT GAC TTG GGT TTG GG	245–264	
CXCL2	FP: ATC CAG AGC TTG AGT GTG ACG C	194–215	NM_009140.2
	RP: AAG GCA AAC TTT TTG ACC GC	264–283	
FOXP3	FP: GGC CCT TCT CCA GGA CAG A	551–570	NM_054039.2
	RP: GCT GAT CAT GGC TGG GTT GT	642–662	
GATA3	FP: AGT CCT CAT CTC TTC ACC TTC C	1027–1048	NM_008091.3
	RP: GGC ACT CTT TCT CAT CTT GCC TG	1116–1138	
RORγt	FP: CCG CTG AGA GGG CTT CAC	75–93	AJ1232394
	RP: TGC AGG AGT AGG CCA CAT TAC	283–304	
T-bet	FP: GCC AGG GAA CCG CTT ATA TG	823–843	AF241242
	RP: GAC GAT CAT CTG GGT CAC ATT GT	935–958	
JEV	FP: GGC TTA GCG CTC ACA TCC A	4132–4150	AB920399.1
	RP: GCT GGC CAC CCT CTC TTC TT	4207–4226	
β-actin	FP: TGG AAT CCT GTG GCA TCC ATG AAA C	885–909	NM_007393.3
	RP: TAA AAC GCA GCT CAG TAA CAG TCC G	1209–1233	

*IL* interleukin, *FP* forward primer, *RP* reverse primer

### Quantitative real-time RT-PCR for determination of viral burden and cytokine expression

Viral burden and the expression of cytokines (IL-1β, IL-6, IL-10, IL-17, IFN-γ) and chemokines (CCL2, CCL3, CCL4, CCL5, CXCL1, CXCL2) in inflammatory and lymphoid tissues were determined using quantitative SYBR Green-based real-time RT-PCR (real-time qRT-PCR). Mice were intraperitoneally (i.p.) infected with JEV (3.0 × 10^7^ pfu). Tissues including brain and spleen were harvested at 3, 4, 5, and 7 dpi following extensive cardiac perfusion with Hank’s balanced salt solution (HBSS). Total RNAs were extracted from tissues using easyBLUE (iNtRON, Inc., Daejeon, Korea). Reverse transcription of total RNAs was performed using High-Capacity cDNA Reverse Transcription Kits (Applied Biosystems, Foster, CA). These complementary DNAs (cDNAs) were used for real-time qPCR using a CFX96 Real-Time PCR Detection system (Bio-Rad Laboratories, Hercules, CA). The reaction mixture contained 2 μl of template cDNA, 10 μl of 2× SYBR Primix Ex Taq, and 200-nM primers at a final volume of 20 μl. The reactions were denatured at 95 °C for 30 s and then subjected to 45 cycles of 95 °C for 5 s and 60 °C for 20 s. After the reaction cycle was completed, the temperature was increased from 65 to 95 °C at a rate of 0.2 °C/15 s, and the fluorescence was measured every 5 s to construct a melting curve. A control sample containing no template DNA was run with each assay, and all determinations were performed at least in duplicates to ensure reproducibility. The authenticity of amplified product was determined by melting curve analysis. Viral RNA burden in infected samples was expressed as viral RNA copies per microgram of RNA. The expression levels of cytokines and chemokines were normalized to β-actin. All data were analyzed using Bio-Rad CFX Manager version 2.1 analysis software (Bio-Rad Laboratories).

### Infiltrated leukocyte analysis in the CNS

Mice infected with JEV were perfused with 30 ml of HBSS at 3, 5, and 7 dpi via cardiac puncture of the left ventricle. The brains were harvested and homogenized by gently pressing them through 100-mesh tissue sieves, after which they were digested with 25 μg/ml of collagenase type IV (Worthington Biochem, Freehold, NJ), 0.1 μg/ml trypsin inhibitor *Nα-p*-tosyl-l-lysine chloromethyl ketone, 10 μg/ml DNase I (Amresco, Solon, OH), and 10 mM HEPE in HBSS at 37 °C for 1 h with shaking. Cells were separated using Optiprep density gradient (18/10/5 %) centrifugation at 800×*g* for 30 min (Axis-Shield, Oslo, Norway), after which cells collected from the 18 to 10 % interface were washed twice with PBS. Cells were then counted and stained for CD11b, Ly6G, Ly6C, CD45, F4/80, CD3, CD4, CD8, and NK1.1 using directly conjugated antibodies (eBioscience) at 4 °C for 30 min. Finally, these cells were fixed with 10 % formaldehyde. Data collection and analysis were performed with a FACS Calibur flow cytometer (Becton Dickson Medical Systems, Sharon, MA) and the FlowJo (Tree Star, San Carlos, CA) software, respectively.

### Analysis and activation of NK cells

The activity of NK cells was assessed by their capacity to produce IFN-γ following brief stimulation with PMA and ionomycin (Sigma-Aldrich). Briefly, splenocytes were prepared from Ccr5^+/+^ and Ccr5^−/−^ mice at 2 dpi and stimulated with PMA and ionomycin (PMA at 50 ng/ml, ionomycin at 750 ng/ml) in the presence of monensin (2 μM) to induce the expression of IFN-γ for 1 h. After stimulation, cells were surface-stained with FITC-anti-mouse-CD3ε, PE-Cy7 anti-mouse NK1.1, biotin-conjugated anti-mouse pan-NK cells (CD49b) [DX5] antibodies, and streptavidin-APC at 4 °C for 30 min. Cells were then washed twice with FACs buffer containing monensin. After fixation, cells were permeabilized with 1× permeabilization buffer (eBioscience) and stained intracellularly with PE anti-mouse IFN-γ (XMF1.2) antibody in permeabilization buffer at room temperature for 30 min. After cells were washed with PBS twice, analysis was performed using a FACS Calibur flow cytometer and FlowJo software.

### JEV-specific CD4^+^ and CD8^+^ T cell responses

To monitor CD4^+^ and CD8^+^ T cell responses specific for JEV, surviving mice were sacrificed at 7 dpi and splenocytes were prepared. Erythrocytes were depleted by treating single-cell suspensions with ammonium chloride-containing Tris buffer (NH_4_Cl-Tris) at 37 °C for 5 min. These splenocytes were then cultured in 96-well culture plates (5 × 10^5^ cells/well) with synthetic peptide epitopes (NS1_132–145_, NS3_563–575_, or NS4B_215–225_) in the presence of anti-CD154-PE for 12 h or for 6 h to evaluate CD4^+^ orCD8^+^ T cell responses, respectively [28, 29]. Monensin (2 μM) was added to the antigen-stimulated cells 6 h before harvest. Cells were washed with PBS twice and surface-stained with FITC-anti-CD4 or CD8 antibodies at 4 °C for 30 min, followed by washing twice with PBS containing monensin. After fixation, cells were washed twice with permeabilization buffer and stained with PE-anti-IFN-γ and APC-anti-TNF-α antibodies in permeabilization buffer at room temperature for 30 min. Finally, cells were washed twice with PBS and fixed using fixation buffer. Samples were analyzed using a FACS Calibur flow cytometer and FlowJo software.

### Intracellular staining for analysis of CD4^+^ Th1, Th17, and Treg cells

To monitor CD4^+^ Th subsets, mice were infected i.p. with 3.0 × 10^7^ pfu of JEV and sacrificed at 3 and 5 dpi. Brain leukocytes and splenocytes were prepared and cultured in 96-well plates (10^6^ cells/well) with PMA/ionomycin (Th1 and Th17) in the presence of monensin (2 μM) at 37 °C for 5 h. Stimulated cells were washed twice with PBS and surface-stained with FITC-anti-CD4 at 4 °C for 30 min. After washing twice with PBS containing monensin and fixation, cells were washed twice with permeabilization buffer (eBioscience, SanDiego, CA) and then stained with PerCP-anti-IFN-γ and APC-anti-IL-17α in permeabilization buffer at room temperature for 30 min. After washing twice with PBS, cells were fixed with fixation buffer. To monitor Treg cells, brain leukocytes and splenocytes were surface-stained with FITC-anti-CD4 markers on ice for 30 min, followed by fixation with permeabilization concentrate buffer (eBioscience, sSan Diego, CA) at 4 °C for 6 h. After fixation, cells were washed twice with permeabilization and stained with PE-anti-Foxp3 in permeabilization buffer at room temperature for 30 min. Sample analysis was performed with a FACS Calibur flow cytometer.

### Purification and trafficking analysis of CCR5^+^CD4^+^ Foxp3^+^ Treg cells

CCR5^+^CD4^+^Fopx3^+^ Treg cells were isolated from the spleen of Foxp3^GFP^ knock-in mice using a FACS Aria sorter (Becton Dickson, Palo Alto, CA) with a final cell purity of ≥95 %. CCR5^+^CD4^+^Fopx3^+^ Treg cells were resuspended at density of 10^7^ cells/ml in RPMI 1640 complete medium containing 10 % FBS, 1 % l-glutamine, 1 % nonessential amino acids, and 1 % penicillin/streptomycin. CCR5^+^CD4^+^Foxp3^+^Treg cells (2 × 10^6^ cells/mouse) were injected intravenously into JEV-infected Ccr5^−/−^ mice at 3 dpi. After injecting donor cells, brain and spleen tissues were harvested at 5 dpi. Infiltrated cells were analyzed for the presence of GFP-labeled cells using a FACS Calibur flow cytometer. CCR5^−^CD4^+^Fopx3^+^ Treg cells were purified from Ccr5^−/−^·Foxp3^GFP^ mice and adoptively transferred into Ccr5^−/−^ mice for the control group to CCR5^+^CD4^+^Foxp3^+^ Treg-recipients. In some experiments, IL-10-producing CCR5^+^CD4^+^Foxp3^GFP^ Tregs were detected by intracellular IL-10 staining combined with surface staining with CCR5 and CD4.

### Statistical analysis

All data are expressed as averages ± standard deviation. Statistically significant differences between groups were analyzed using an unpaired two-tailed Student’s *t* test for leukocyte population analysis and in vitro experiments or ANOVA and post hoc testing for multiple comparisons of the means. The significance of differences in viral burden and in vivo cytokine gene expression was evaluated by Mann-Whitney test or unpaired two-tailed Student’s *t* test. Kaplan-Meier survival curves were analyzed using the log-rank test. A *p* value ≤0.05 was considered to indicate statistical significance. All data were analyzed using the Prism software (GraphPad Prism 4, San Diego, CA).

## Results

### CCR5 is essential for protection against JE but dispensable for control of viral replication

**Fig. 1:**
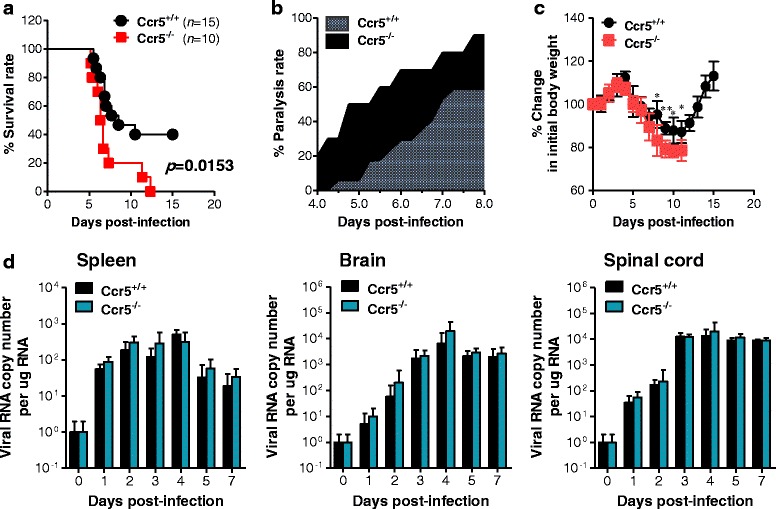
CCR5 is essential for protection against JE but dispensable for control of JEV replication. **a** Susceptibility of CCR5-ablated mice to JE. Ccr5^+/+^ and Ccr5^−/−^ mice (4 to 6 weeks old, *n* = 10–15) was inoculated i.p. with JEV (3.0 × 10^7^ pfu). The survival rate was examined over 15 days. **b** Ratio of mice showing neurological disorders during JE progression. Mice infected with JEV were examined every 6 h from 4 to 8 dpi. **c** Changes in body weight. Data are average percentages ± SD of body weight relative to that at the time of challenge. **d** Viral burden in lymphoid and inflammatory tissues during JE. Viral burden in lymphoid (spleen) and inflammatory tissues (brain and spinal cord) of infected mice (*n* = 5–6) were assessed by real-time qRT-PCR at the indicated time points. Viral RNA load was expressed as viral RNA copy number per microgram of total RNA (*n* = 5–7). *p < 0.05; **p < 0.01 compared to the levels in the corresponding groups

The chemokine receptor CCR5 is believed to play a critical role in recovery from flavivirus encephalitis via efficient leukocyte trafficking to the brain [16–18]. CCR5 is also a key mediator to recruit CD4^+^Foxp3^+^ Tregs known as regulatory CD4^+^ T cell subset to dampen exacerbated inflammation such as viral encephalitis [21–24]. Although the role of CD4^+^Foxp4^+^ Tregs in flavivirus encephalitis remains elusive, CCR5-dependent recruitment of CD4^+^Foxp3^+^ Tregs may play certain roles in the control of encephalitis progression caused by flavivirus infection. To address this issue of CCR5 in flavivirus encephalitis, we first confirmed the role of CCR5 in JE progression using CCR5-deficient (Ccr5^−/−^) mice. After Ccr5^+/+^ and Ccr5^−/−^ mice were infected with JEV, surviving mice were monitored until 15 dpi (Fig. 1a). Mice in both groups showed similar clinical signs, starting with generalized piloerection, paresis, and rigidity and followed by progression into severe neurological signs such as postural imbalance, ataxia, and generalized tonic-clonic seizure from 4 to 6 dpi. However, CCR5 ablation resulted in marked increases in mortality after showing neurological disorders, with a mortality rate of 100 % for Ccr5^−/−^ mice vs. 54 % for Ccr5^+/+^ mice after JEV infection (3.0 × 10^7^ pfu). Likewise, Ccr5^−/−^ mice showed a rapid increase in the frequency of neurological disorder starting from 3 to 4 dpi after JEV infection (3.0 × 10^7^ pfu) with greater body weight loss, compared to Ccr5^+/+^ mice (Fig. 1b, c). However, the viral burden in the extraneural lymphoid tissue (spleen) and CNS (brain and spinal cord) of Ccr5^−/−^ mice was not increased compared to that of Ccr5^+/+^ mice (Fig. 1d). Therefore, these results indicate that CCR5 ablation could result in an increased susceptibility to JE progression irrespective of viral replication.

### CCR5 ameliorates JE progression

**Fig. 2:**
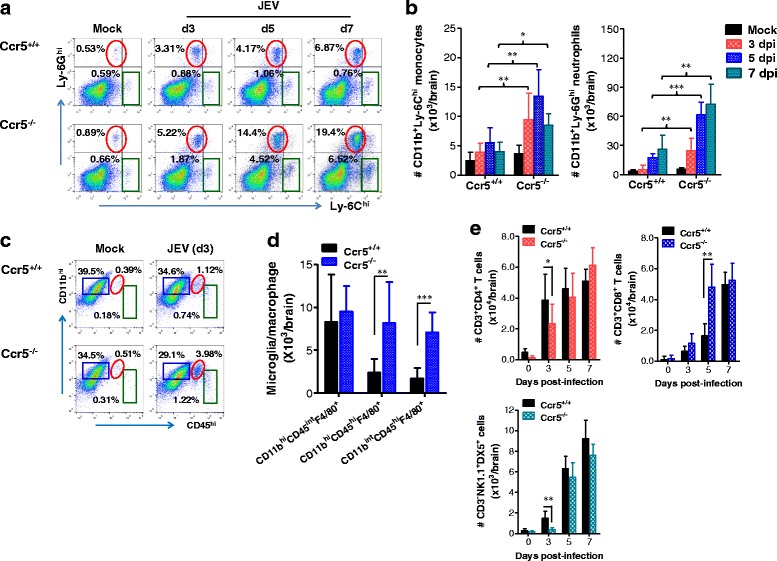
CCR5 regulates JE progression by altering the infiltration of leukocytes in the brain. **a**, **b** The frequency and number of Ly-6C^hi^ monocytes and Ly-6G^hi^ granulocytes in the brain. Ccr5^+/+^ and Ccr5^−/−^ mice were inoculated i.p. with JEV (3.0 × 10^7^ pfu), and the frequency (**a**) and total number (**b**) of Ly-6C^hi^ monocytes and Ly-6G^hi^ granulocytes in the CNS were determined by flow cytometric analysis at 3, 5, and 7 dpi using vigorous heart perfusion. Values in representative dot-plots denote the average percentage of the indicated population after gating on CD11b^+^ cells. **c**, **d** Resting and activated microglia/macrophage number in the CNS. The number of resting (CD11c^−^CD11b^hi^CD45^int^F4/80^+^) and activated (CD11c^-^CD11b^hi^CD45^hi^ F4/80^+^) as well as other myeloid-derived leukocytes (CD11b^int^CD45^hi^ F4/80^+^) was enumerated using flow cytometric analysis at 3 dpi. Values in representative dot-plots denote the average percentage of the indicated population after gating on CD11c^−^F4/80^+^ cells. **e** Accumulated number of NK cells, CD4^+^, and CD8^+^ T cells in the CNS. Total accumulated number of NK cells (CD3^−^NK1.1^+^DX5^+^), CD4^+^ (CD3^+^CD4^+^), and CD8^+^ (CD3^+^CD8^+^) T cells in the CNS were enumerated using flow cytometric analysis at 3, 5, and 7 dpi. Data are averages ± SD of the indicated cell populations derived from at least three independent experiments (*n* = 4–5). *p < 0.05; **p < 0.01; ***p < 0.001 compared with the levels of the indicated groups

To further characterize CNS inflammation caused by JEV infection in Ccr5^+/+^ and Ccr5^−/−^ mice, we assessed the infiltration of CD11b^+^Ly-6C^hi^ monocytes and CD11b^+^Ly-6G^hi^ granulocytes into CNS, because infiltration of these cell populations derived from the myeloid cell lineage has been used to evaluate CNS inflammation [30]. Our results revealed that a markedly higher frequency of infiltrated CD11b^+^Ly-6C^hi^ monocytes and CD11b^+^Ly-6G^hi^ granulocytes was retained in the brain of Ccr5^−/−^ mice at 3, 5, and 7 dpi, compared to that in the brain of Ccr5^+/+^ mice (Fig. 2a). Similarly, the absolute number of CD11b^+^Ly-6C^hi^ monocytes and CD11b^+^Ly-6G^hi^ granulocytes infiltrating the brain of Ccr5^−/−^ mice was increased two- and threefold at 5 dpi, respectively (Fig. 2b). Moreover, microglia cells contribute to the progression of encephalitis caused by some neurotropic viruses, such as WNV [31, 32]. Thus, four-color (CD11c/CD11b/CD45/F4/80) staining was used to distinguish resting from activated microglia. Based on the CNS myeloid cell classification method of Ford et al. [33], equivalent percentages and similar absolute numbers of resting microglia (CD11c^−^CD11b^hi^CD45^int^F4/80^+^) were detected in both Ccr5^+/+^ and Ccr5^−/−^ mice. However, activated microglia/macrophages (CD11c^−^CD11b^hi^CD45^hi^F4/80^+^) and other myeloid-derived leukocytes (CD11c^−^CD11b^int^CD45^hi^F4/80^+^) in the brain of Ccr5^−/−^ mice were detected at higher frequencies and absolute numbers compared to the brain of Ccr5^+/+^ mice (Fig. 2c, d). These results indicate that CCR5 ablation could exacerbate JE by enhancing the accumulation of inflammatory monocytes and granulocytes in the CNS, along with activation of microglia. CCR5 is also considered to be involved in the recruitment of lymphoid lineage-derived cells, including CD4^+^, CD8^+^ T cells, and NK cells [34, 35], which may play a beneficial role in the control of JE progression [36–38]. To better understand CNS inflammation in Ccr5^−/−^ mice following JEV infection, CD4^+^ and CD8^+^ T cells, and NK cells were enumerated in the brain. Infiltration of both CD4^+^ and NK cells was evidenced by a transient increase in Ccr5^+/+^ mice at 3 dpi, after which the total number of CD4^+^ and NK cells in Ccr5^+/+^ and Ccr5^−/−^ mice was comparable at 5 and 7 dpi. This result implies that CD4^+^ T and NK cells might not predominate in the control of JE that has already progressed, because infected mice usually showed clinical signs at around 4–5 dpi. However, CD8^+^ T cells infiltrated the brain of Ccr5^−/−^ mice at gradually increased levels up to 5 dpi compared to that in the brain of Ccr5^+/+^ mice (Fig. 2e), indicating that enhanced infiltration of CD8^+^ T cells is closely associated with JE progression.

**Fig. 3:**
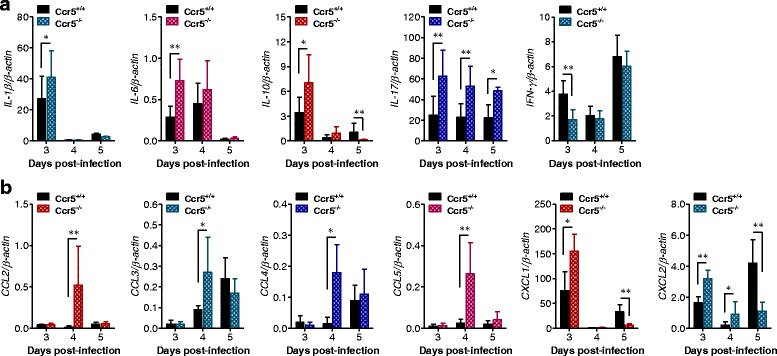
The dynamic expression of cytokines and chemokines in CCR5-ablated mice, depending on JE progression. **a** Expression of pro- and anti-inflammatory cytokines in the CNS. **b** The expression of CC and CXC chemokines in the CNS. The expression of cytokines and chemokines in the CNS was determined by real-time qRT-PCR at the indicated time points after Ccr5^+/+^ and Ccr5^−/−^ mice were inoculated i.p. with JEV (3.0 × 10^7^ pfu). Data are averages ± SD of β-actin-normalized cytokine expression derived from at least three independent experiments (*n* = 4–5). *p < 0.05; **p < 0.01 compared with the levels of the indicated groups

In terms of CNS inflammation, the expression of cytokines and chemokines within the CNS is required for encephalitis, because encephalitis caused by neurotropic viruses is indirectly derived from CNS degeneration, due to robust immunological responses such as uncontrolled secretion of cytokines and chemokines, which results in the activation of microglia and astrocytes [10, 11]. Therefore, we examined the expression of cytokines and chemokines in the CNS. We found that CCR5 ablation resulted in early and increased expression of pro- and anti-inflammatory cytokines at 3 dpi. However, IFN-γ expression was higher in Ccr5^+/+^ mice at 3 dpi, compared to in Ccr5^−/−^ mice. The early increase in CNS infiltration of CD4^+^ and NK cells likely led to earlier and higher expression of IFN-γ. In addition, it was interesting that the expression levels of some cytokines were reversed at 5 dpi (Fig. 3a). Notably, the anti-inflammatory cytokine IL-10 was expressed at higher levels in Ccr5^+/+^ mice at 5 dpi, whereas IL-17, which is produced by CD4^+^ Th17 cells, was expressed at a lower level in Ccr5^+/+^ mice compared to those in Ccr5^−/−^ mice at 5 dpi. With regard to chemokine expression, CC chemokines were expressed at higher levels in Ccr5^−/−^ mice 4 dpi compared to those in Ccr5^+/+^ mice. Interestingly, the expression levels of CXC chemokines, CXCL1 and CXCL2, were enhanced in Ccr5^−/−^ mice at 3 dpi, and such expression was reversed at 5 dpi (Fig. 3b). Collectively, these results indicate that the expression levels of pro-/anti-inflammatory cytokines and CC/CXC chemokines in the CNS of Ccr5^+/+^ and Ccr5^−/−^ mice could change dynamically depending on the progression of JE.

### Kinetic analysis of myeloid and lymphoid cells in the spleen and blood of CCR5-ablated mice

**Fig. 4:**
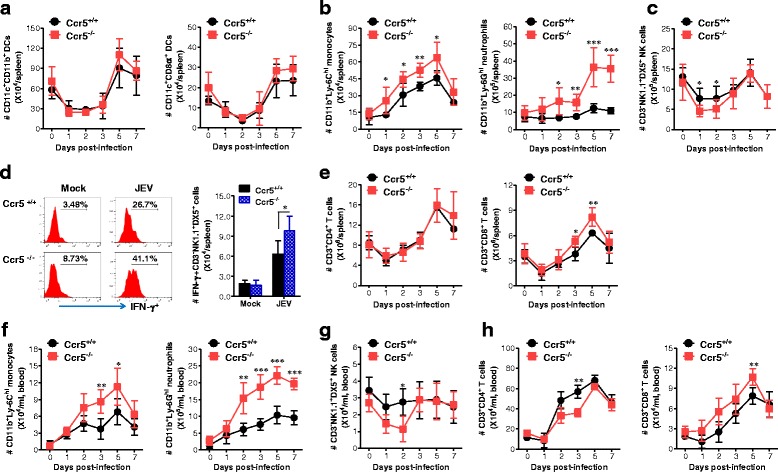
Kinetic analysis of DC subpopulations, myeloid cells, NK cells, and CD4/CD8 T cells in the spleen and blood of CCR5-ablated mice. **a–c** Kinetic analysis of DC subpopulations, Ly-6C^hi^ monocytes, Ly-6G^hi^ neutrophils, and NK cells in the spleen. Ccr5^+/+^ and Ccr5^−/−^ mice were inoculated i.p. with JEV (3.0 × 10^7^ pfu), and the absolute numbers of splenic DC subpopulations (CD11c^+^CD11b^+^ myeloid DC and CD11c^+^CD8α^+^ lymphoid DC) (**a**), Ly-6C^hi^ monocytes and Ly-6G^hi^ neutrophils (**b**), and NK cells (**c**) were determined using flow cytometric analysis at the indicated time points. **d** Activation of NK cells. The activation of CD3^−^NK1.1^+^DX5^+^ NK cells was evaluated by intracellular IFN-γ staining upon stimulation with PMA plus ionomycin. Values in the histograms on the left denote the average percentages of IFN-γ-producing cells after gating on CD3^−^NK1.1^+^DX5^+^ NK cells. **e** Absolute number of CD4^+^ and CD8^+^ T cells in the spleen. Splenic CD4^+^ and CD8^+^ T cells were enumerated from 1 to 7 dpi. **f–h** Kinetic analysis of myeloid, NK, and T cells in the blood of Ccr5^+/+^ and Ccr5^−/−^ mice. The numbers of myeloid cells (Ly-6C^hi^ monocytes and Ly-6G^hi^ neutrophils) (**f**), NK cells (**g**), and CD4^+^/CD8^+^ T cells (**h**) were determined using flow cytometric analysis at the indicated time points. Data are averages ± SD of values derived from at least three independent experiments (*n* = 3–5). *p < 0.05; **p < 0.01; ***p < 0.001 compared to the levels in the corresponding groups

Because the CNS expression pattern of CC and CXC chemokines in Ccr5^+/+^ and Ccr5^−/−^ mice differed according to JE progression, this CC/CXC chemokine expression may affect the migration of myeloid and lymphoid cells, including monocytes, granulocytes, NK, and T cells in both Ccr5^+/+^ and Ccr5^−/−^ mice. Therefore, to better understand the recruitment of myeloid and lymphoid cells in the CNS, we kinetically examined the number of myeloid and lymphoid cells in the spleen and blood of Ccr5^+/+^ and Ccr5^−/−^ mice depending on JE progression. In addition, analyzing the spleen could provide insight into how CCR5 modulates innate and inflammatory responses immediately after infection. Analysis of myeloid CD11b^+^ and lymphoid CD8α^+^ DC subsets revealed that both Ccr5^+/+^ and Ccr5^−/−^ mice exhibited a similar reduction in the spleen (Fig. 4a), as wild-type mice have been previously shown to have a transiently decreased number of myeloid and lymphoid DCs due to JEV infection [27]. However, Ccr5^−/−^ mice had higher numbers of inflammatory CD11b^+^Ly-6C^hi^ monocytes and Ly-6G^hi^ neutrophil in the spleen up to 5 dpi, compared to Ccr5^+/+^ mice (Fig. 4b), indicating that Ccr5^−/−^ mice experienced a severe inflammatory reaction in the spleen. Also, the absolute number of splenic CD3^-^NK1.1^+^DX5^+^ NK cells was transiently decreased in both Ccr5^+/+^ and Ccr5^−/−^ mice, but Ccr5^+/+^ mice had a higher number of CD3^−^NK1.1^+^DX5^+^ NK cells in the spleen at the early phase (1 and 2 dpi), compared to Ccr5^−/−^ mice (Fig. 4c). Interestingly, our results revealed that the frequency and number of NK cells producing IFN-γ were increased in Ccr5^−/−^ mice, rather than in Ccr5^+/+^ mice, when the activation of NK cells was evaluated by assessing their production of IFN-γ (Fig. 4d). These data indicate that NK cells might not be involved in the amelioration of JE in Ccr5^+/+^ mice. Furthermore, our results revealed that the number of Ly-6C^hi^ monocytes and Ly-6G^hi^ neutrophils in the blood followed the infiltration trends of Ly-6C^hi^ monocytes and Ly-6G^hi^ neutrophils in the brain. CCR5-ablated mice had higher numbers of Ly-6C^hi^ monocytes and Ly-6G^hi^ neutrophils in the blood up to 5 dpi, compared to Ccr5^−/−^ mice (Fig. 4f). Also, NK cells in the blood of Ccr5^−/−^ mice were detected at transiently lower levels at the early phase (1 and 2 dpi), after which they were comparable in Ccr5^+/+^ and Ccr5^−/−^ mice (Fig. 4g). CD4^+^ T cell numbers were transiently higher number in the blood of Ccr5^+/+^ mice than in Ccr5^−/−^ mice at 2 and 3 dpi, but CD8^+^ T cells accumulated to higher levels in Ccr5^−/−^ mice, rather than Ccr5^+/+^ mice, at up to 5 dpi (Fig. 4h). Therefore, these results suggest that CCR5 ablation is not involved in the migration of myeloid and lymphoid cells from the blood into the brain during JE progression.

### Adaptive T cell immune responses in CCR5-ablated mice

**Fig. 5:**
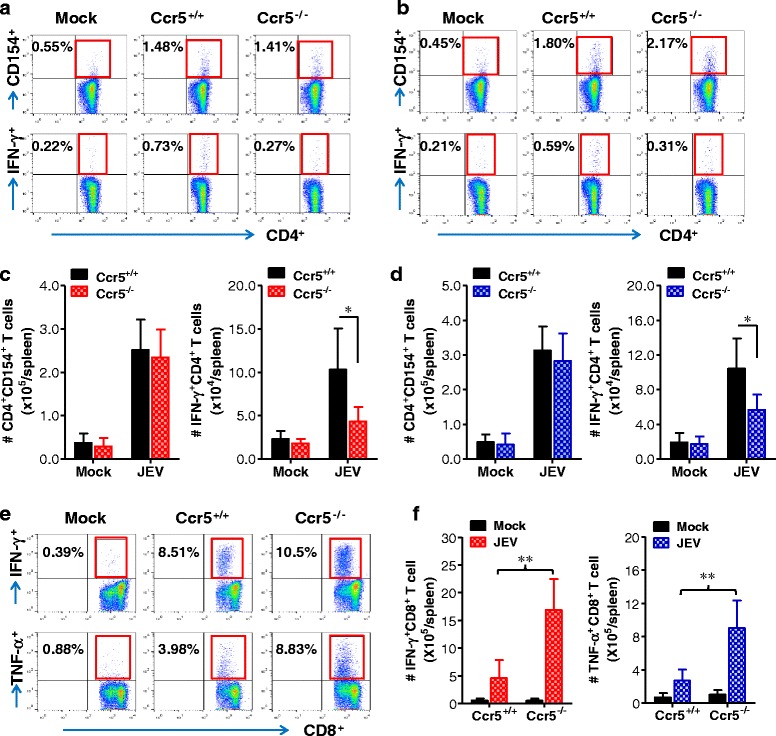
Adaptive T cell responses of CCR5-ablated mice following JEV infection. **a–d** JEV-specific CD4^+^ T cell responses. **e**, **f** JEV-specific CD8^+^ T cell responses. Splenocytes prepared from surviving mice at 7 days following JEV (3.0 × 10^7^ pfu) infection were stimulated with JEV epitope peptide of CD4^+^ T cells (**a**, **c** NS1_132–145_; **b**, **d**, NS3_563–574_) and of CD8^+^ T cells (**e**, **f** NS4B_215–223_) for 12 and 6 h, respectively. The frequency and absolute number of JEV-specific CD4^+^ and CD8^+^ T cells were evaluated by intracellular CD154 and cytokine (IFN-γ or TNF-α) staining combined with CD4 and CD8 surface staining. Values in representative dot-plots are the average percentage of the indicated cell population, while bar charts show the average ± SD of values derived from at least three independent experiments (*n* = 3–4). *p < 0.05; **p < 0.01 compared with the levels of the indicated groups

Antiviral adaptive immune responses, including those mediated by effector antigen-specific CD4^+^ and CD8^+^ T cells, are required for the regulation of JE progression through the control and clearance of JEV in extraneural lymphoid tissues and the CNS [36–38]. Although both Ccr5^+/+^ and Ccr5^−/−^ mice infected with JEV exhibited neurological disorders at 4–5 dpi, which is before functional adaptive immune responses were fully induced, we examined the generation of JEV-specific CD4^+^ T cell responses in surviving Ccr5^+/+^ and Ccr5^−/−^ mice at 7 dpi using intracellular CD154 staining combined with intracellular cytokine IFN-γ staining, because CD154^+^CD4^+^ T cells could enable us to enumerate viable JEV-specific CD4^+^ T cells in response to stimulation with epitope peptides [28, 29]. As shown in Fig. 5a, b, similar levels of JEV-specific CD154^+^CD4^+^ T cells were detected in Ccr5^+/+^ and Ccr5^−/−^ mice. However, the frequencies of JEV-specific CD4^+^ T cells producing IFN-γ were higher in Ccr5^+/+^ mice upon stimulation with the epitope peptides NS1_132–145_ and NS3_563–574_, compared to those in Ccr5^−/−^ mice. Also, IFN-γ^+^CD4^+^ T cell numbers were higher in the spleen of Ccr5^+/+^ mice upon stimulation with JEV epitope peptides at 7 dpi, compared to Ccr5^−/−^ mice (Fig. 5c, d). Presumably, this increase in IFN-γ^+^CD4^+^ T cells specific for JEV Ag may contribute in part to the control of JE progression in Ccr5^+/+^ mice at a later phase. In contrast, the frequency and total number of JEV-specific CD8^+^ T cells producing IFN-γ and TNF-α in response to stimulation with the CD8^+^ T cell epitope NS4B_215–223_ were higher in Ccr5^−/−^ mice than in Ccr5^+/+^ mice (Fig. 5e, f). This result was inconsistent with the enhanced CNS infiltration of CD8^+^ T cells. Taken together, these results suggest that antiviral JEV-specific CD8^+^ T cells may not be key players in the control of JE progression in Ccr5^+/+^ mice at the early phase (4–5 dpi). However, IFN-γ^+^CD4^+^ T cells specific for JEV Ag appear to play a role in the control of JE progression in Ccr5^+/+^ mice at the later phase (7–8 dpi).

### Skewed IL-17^+^CD4^+^ Th17 responses of CCR5-ablated mice during JE progression

**Fig. 6:**
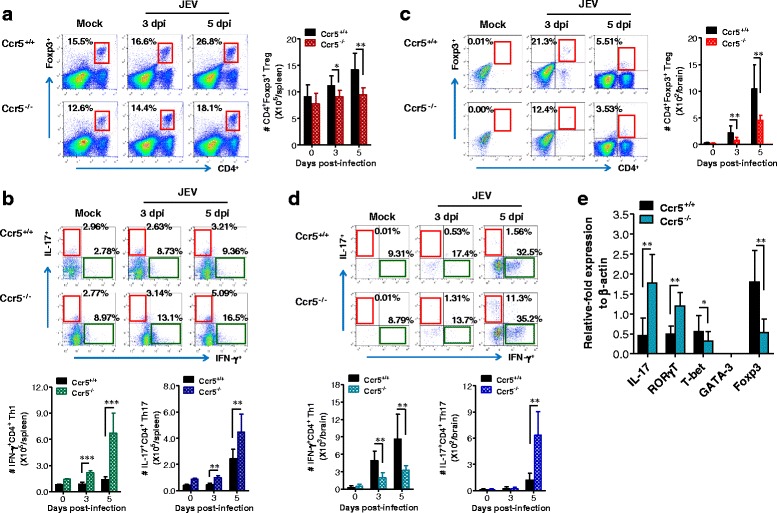
Early skewed IL-17^+^CD4^+^ Th17 response of CCR5-ablated mice during JE progression. **a**, **b** Frequency and number of CD4^+^Foxp3^+^ Tregs, IFN-γ^+^CD4^+^ Th1, and IL-17^+^CD4^+^ Th17 cells in the spleen of CCR5-ablated mice. **c**, **d** Frequency and number of CD4^+^Foxp3^+^ Tregs, IFN-γ^+^CD4^+^ Th1, and IL-17^+^CD4^+^ Th17 cells in the brain of CCR5-ablated mice. The frequency and absolute number of CD4^+^Foxp3^+^ Tregs (**a**, **c**), IFN-γ^+^CD4^+^ Th1, and IL-17^+^CD4^+^ Th17 cells (**b**, **d**) in the spleen (**a**, **b**) and brain (**c**, **d**) of Ccr5^+/+^ and Ccr5^−/−^ mice were determined by flow cytometric analysis at 3 and 5 days following JEV (3.0 × 10^7^ pfu) infection. CD4^+^Foxp3^+^ Tregs were detected with intracellular Foxp3 and surface CD4 staining, and the frequency and number of IFN-γ^+^CD4^+^ Th1 and IL-17^+^CD4^+^ Th17 cells were determined by intracellular cytokine staining in response to PMA + ionomycin stimulation of splenocytes or brain leukocytes prepared from Ccr5^+/+^ and Ccr5^−/−^ mice. Values in representative dot-plots are the average percentage of Foxp3^+^ cells, IFN-γ^+^ and IL-17^+^ in CD4^+^ T cells. **e** Expression of transcription factors by CNS-infiltrated CD4^+^ T cells. After vigorous heart perfusion, sorted CD4^+^ T cells from CNS-infiltrated leukocytes were briefly stimulated with PMA plus ionomycin for 3 h. The expression of transcription factors of CD4^+^ Th1, Th2, Th17, and Tregs was determined by real-time qRT-PCR using total RNA extracted from stimulated CD4^+^ T cells. Data are averages ± SD of values derived from at least three independent experiments (*n* = 3–4). *p < 0.05; **p < 0.01; ***p < 0.001 compared with the levels of the indicated groups

NK and CD8^+^ T cells did not appear to play a dominant regulatory function in already progressed JE because Ccr5^+/+^ mice failed to show enhanced CNS infiltration of NK or CD8^+^ T cells at 5 dpi, compared to Ccr5^−/−^ mice. In addition, a stronger JEV-specific CD8^+^ T cell response was elicited in Ccr5^−/−^ mice at 7 dpi, rather than Ccr5^+/+^ mice. Although the IFN-γ^+^CD4^+^ Th1 response specific for JEV Ag was stronger in Ccr5^+/+^ mice, adaptive JEV-specific CD4^+^ T cell responses take some time to develop. Therefore, adaptive JEV-specific CD4^+^ T cell responses may contribute to the control of JE progression only at a later stage. In our results, dynamic changes in the expression of pro-/anti-inflammatory cytokines, particularly IL-10 and IL-17, were observed in the CNS during JE progression at 3 and 5 dpi. Therefore, we evaluated the dynamic response of CD4^+^ Th subsets that produce typical pro- or anti-inflammatory cytokines: CD4^+^ Th1 expressing IFN-γ, CD4^+^ Th17 expressing IL-17, and CD4^+^Foxp3^+^ Tregs expressing IL-10. First, we examined the frequency and total number of CD4^+^Foxp3^+^ Tregs in the spleens of Ccr5^+/+^ and Ccr5^−/−^ mice at 3 and 5 dpi, a time point at which the mice showed dynamic changes in cytokine expression. Ccr5^+/+^ mice exhibited a significantly higher frequency and number of CD4^+^Foxp3^+^ Tregs at both 3 and 5 dpi, compared to Ccr5^−/−^ mice (Fig. 6a). However, Ccr5^−/−^ mice showed an increased frequency and total number of CD4^+^ Th1 expressing IFN-γ and CD4^+^ Th17 expressing IL-17 in the spleen at both 3 and 5 dpi, compared to Ccr5^+/+^ mice (Fig. 6b). This result indicates that Ccr5^−/−^ mice had a skewed response of these CD4^+^ Th subsets at the early stage of JE progression. To further define the skewed response of the CD4^+^ Th subsets in Ccr5^−/−^ mice, we examined CNS-infiltrated CD4^+^ Th subsets at 3 and 5 dpi during JE progression. Ccr5^+/+^ mice showed a rapidly increased frequency and absolute number of CNS-infiltrated CD4^+^Foxp3^+^ Tregs; levels were two- to threefold higher than those of Ccr5^−/−^ mice (Fig. 6c). Also, Ccr5^+/+^ mice exhibited a moderately increased number, but not frequency, of CD4^+^ Th1 expressing IFN-γ in the CNS at 3 and 5 dpi, whereas a markedly increased frequency and number of CD4^+^ Th17 expressing IL-17 were detected in the CNS of Ccr5^−/−^ mice; being approximately tenfold higher than those of Ccr5^+/+^ mice (Fig. 6d). Moreover, CD4^+^ T cells sorted from the CNS of Ccr5^+/+^ mice showed higher expression of the transcription factors T-bet and Foxp3, which are involved in the differentiation of CD4^+^ Th1 cells and Tregs, compared to those from the CNS of Ccr5^−/−^ mice (Fig. 6e). In contrast, CD4^+^ T cells sorted from the CNS of Ccr5^−/−^ mice had higher expression of the CD4^+^ Th17 transcription factor RORγT and IL-17, compared to Ccr5^+/+^ mice. These results suggest that CCR5 ablation results in a skewed IL-17^+^CD4^+^ Th17 response in both extraneural lymphoid tissue and the CNS, which leads to reduced CNS infiltration of CD4^+^Foxp3^+^ Tregs during JE progression, which is closely associated with exacerbation of JE in Ccr5^−/−^ mice.

### CCR5^+^CD4^+^Foxp3^+^ Tregs ameliorate JE progression in CCR5-ablated mice

**Fig. 7:**
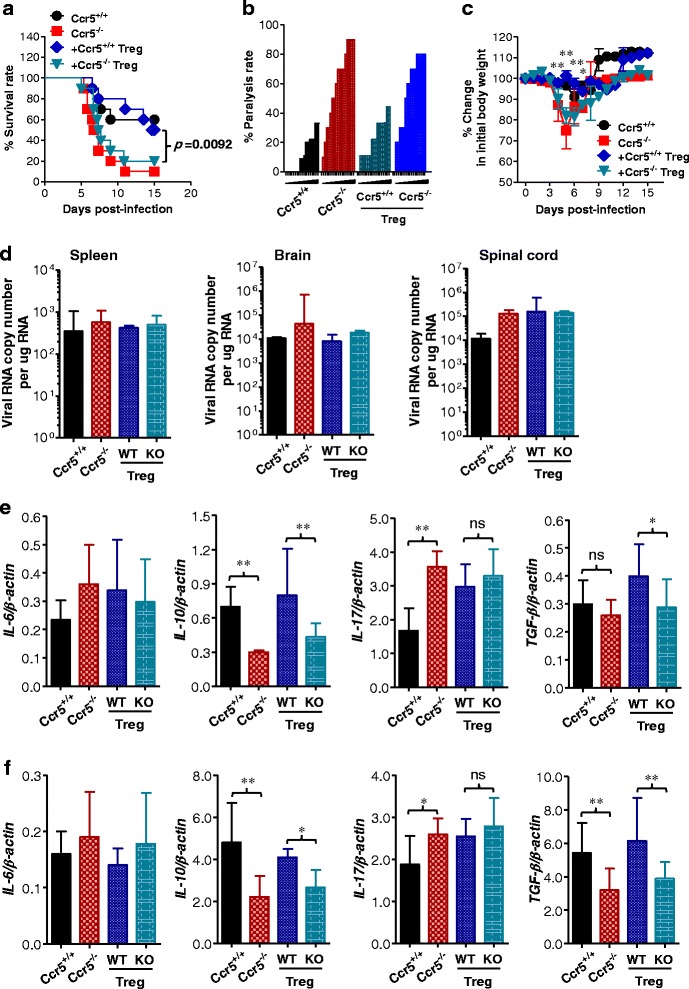
CCR5^+^CD4^+^Foxp3^+^ Tregs ameliorate JE in CCR5-ablated mice. **a** Susceptibility of CCR5-ablated mice to JE following adoptive transfer of CCR5^+^CD4^+^Foxp3^+^ Tregs. CCR5^+^CD4^+^Foxp3^+^ and CCR5^−^CD4^+^Foxp3^+^ Treg cells were purified from Ccr5^+/+.^Foxp3^GFP^ and Ccr5^−/−.^Foxp3^GFP^ mice and adoptively transferred to Ccr5^−/−^ mice (*n* = 10) at 2 days following JEV (3.0 × 10^7^ pfu) infection, respectively. Surviving recipient mice were examined daily. Ccr5^+/+^ and Ccr5^−/−^ mice that did not receive Tregs were used as positive and negative controls, respectively. **b** Ratio of mice showing neurological disorders in CCR5^+^ Treg-injected CCR5-ablated mice. Ccr5^−/−^ recipients that received CCR5^+^ or CCR5^−^ Tregs were examined every 6 h from 4 to 11 dpi. **c** Changes in body weight of CCR5^+^ Treg-injected CCR5-ablated mice during JE. Data are averages ± SD of body weight relative to the time of challenge. **d** Viral burden in lymphoid tissue and CNS of Treg-injected Ccr5^−/−^ recipient. Viral burden in the spleen, brain, and spinal cord of Ccr5^−/−^ recipients was assessed by real-time qRT-PCR at 5 dpi. The viral RNA load was expressed as viral RNA copy number per microgram of total RNA. **e**, **f** The expression of pro- and anti-inflammatory cytokines in lymphoid tissue and CNS of Treg-injected Ccr5^−/−^ recipients. The expression of pro- and anti-inflammatory cytokines in the brain (**e**) and spleen (**f**) of Treg-injected Ccr5^−/−^ recipients was determined by real-time qRT-PCR at 5 dpi. Data are averages ± SD of values derived from at least three independent experiments (*n* = 5–6). *p < 0.05; **p < 0.01 compared with the levels of the indicated groups

Although our results suggest that the increased number of CD4^+^Foxp3^+^ Tregs in extraneural lymphoid tissue and the CNS of Ccr5^+/+^ mice is associated with mild JE, we did not provide direct evidence regarding whether the enhanced response of CD4^+^Foxp3^+^ Tregs plays a beneficial role in JE progression. To address this issue, CCR5^+^CD4^+^Foxp3^+^ Tregs purified from Ccr5^+/+^ mice were injected i.v. into Ccr5^−/−^ mice at 3 dpi, and the recipient mice were examined in terms of mortality and clinical signs up to 15 dpi. As shown in Fig. 7a, Ccr5^−/−^ recipients of CCR5^+^CD4^+^Foxp3^+^ Tregs showed a reduced mortality rate (around 50 %) comparable to that of Ccr5^+/+^ mice. However, Ccr5^−/−^ mice that received CCR5^−^CD4^+^Foxp3^+^ Tregs purified from Ccr5^−/−.^Foxp3^GFP^ mice showed high susceptibility to JE, with a mortality rate of 90 %, similar to that of Ccr5^−/−^ mice. In addition, a reduced proportion of mice showing neurological disorders was observed in Ccr5^−/−^ recipients of CCR5^+^CD4^+^Foxp3^+^ Tregs, even though Ccr5^−/−^ recipients of CCR5^+^CD4^+^Foxp3^+^ Tregs exhibited clinical signs starting at a similar time post-infection to those of Ccr5^−/−^ mice and CCR5^−^CD4^+^Foxp3^+^ Treg recipients (Fig. 7b). Also, Ccr5^−/−^ recipients of CCR5^+^CD4^+^Foxp3^+^ Tregs showed no apparent reduction in body weight during JE progression compared to Ccr5^−/−^ mice receiving no Tregs (Fig. 7c). These results suggest that CCR5^+^CD4^+^Foxp3^+^ Tregs purified from Ccr5^+/+^·Foxp3^GFP^ mice could ameliorate JE progression in Ccr5^−/−^ mice, in contrast with CCR5^-^CD4^+^Foxp3 Tregs purified from Ccr5^−/−.^Foxp3^GFP^ mice. To better understand JE regulation in Ccr5^−/−^ mice after injection with CCR5^+^CD4^+^Foxp3^+^ Tregs, the viral burden in extraneural lymphoid tissues and the CNS was determined at 5 dpi. Our results revealed that injection of CCR5^+^CD4^+^Foxp3^+^ Tregs resulted in no change in the viral burden in Ccr5^−/−^ recipients. This result indicates that CCR5^+^CD4^+^Foxp3^+^ Tregs could regulate JE progression without changing the viral burden (Fig. 7d). Instead, Ccr5^−/−^ recipients of CCR5^+^CD4^+^Foxp3^+^ Tregs showed enhanced expression levels of anti-inflammatory cytokines, including IL-10 and TGF-β, in the brain and spleen at 5 dpi, compared to Ccr5^−/−^ mice receiving no Tregs. However, IL-17 expression was not changed in the brain and the spleen of CCR5^+^CD4^+^Foxp3^+^ Treg-injected Ccr5^−/−^ recipients (Fig. 7e, f). Collectively, these results indicate that CCR5^+^CD4^+^Foxp3^+^ Tregs injected into Ccr5^−/−^ mice ameliorate JE progression by enhancing the expression of anti-inflammatory cytokines.

### CCR5^+^CD4^+^Foxp3^GFP^ Tregs produce IL-10 to ameliorate JE without altering CNS infiltration of Ly-6C^hi^ monocytes, CD4^+^ Th1, or Th17

**Fig. 8:**
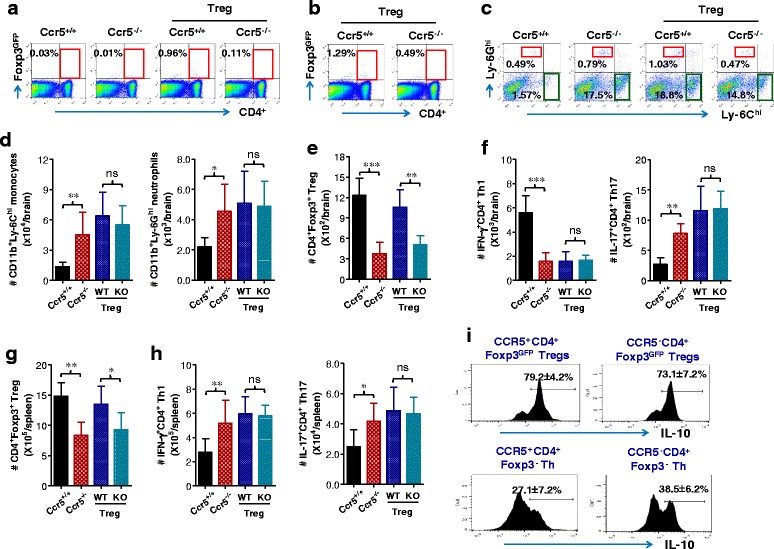
CCR5^+^CD4^+^Foxp3^+^ Tregs ameliorate JE via IL-10 production without affecting the accumulation of myeloid-derived leukocytes, CD4^+^ Th1, or Th17. **a**, **b** Detection of CCR5^+^CD4^+^Foxp3^GFP^ Tregs in the spleen and brain. Adoptively transferred CCR5^+^CD4^+^Foxp3^GFP^ and CCR5^−^CD4^+^Foxp3^GFP^ Tregs were detected by flow cytometry in the spleen (**a**) and brain (**b**) at 5 days following JEV (3.0 × 10^7^ pfu) infection. Values in representative dot-plots denote the average percentages of CCR5^+^CD4^+^Foxp3^GFP^ Tregs in CD4^+^ T cells. **c**, **d** The frequency and number of Ly-6C^hi^ monocytes and Ly-6G^hi^ granulocytes in the CNS of CCR5^+^ or CCR5^−^ Treg-injected CCR5-ablated mice. The frequency (**c**) and number (**d**) of Ly-6C^hi^ monocytes and Ly-6G^hi^ granulocytes in the CNS of CCR5^+^ (WT) or CCR5^−^ (KO) Treg-injected CCR5-ablated mice were determined by flow cytometric analysis at 5 dpi using vigorous heart perfusion. Values in representative dot-plots denote the average percentage of the indicated cell population after gating on CD11b^+^ cells. **e**–**h** Accumulated number of CD4^+^ Th1, Th17, and Tregs in the CNS of CCR5^+^ (WT) or CCR5^−^ (KO) Treg-injected CCR5-ablated mice. The absolute number of CD4^+^ Th1, Th17, and Tregs in the CNS and spleen of Treg-injected Ccr5^−/−^ recipients was determined at 5 dpi. **e** CD4^+^Foxp3^+^ Tregs in brain. **f** CD4^+^ Th1 and Th17 in the brain. **g** CD4^+^Foxp3^+^ Tregs in the spleen. **h** CD4^+^ Th1 and Th17 in the spleen. **i** IL-10 expression in adoptively transferred CCR5^+^CD4^+^Foxp3^GFP^ and CCR5^−^CD4^+^Foxp3^GFP^ Tregs. The expression of IL-10 in CNS-infiltrated CCR5^+^CD4^+^Foxp3^GFP^ and CCR5^−^CD4^+^Foxp3^GFP^ Tregs was evaluated by intracellular IL-10 staining at 5 dpi. Data are averages ± SD of values derived from at least three independent experiments (*n* = 3–4). *p < 0.05; **p < 0.01; *p* < 0.001 compared with the levels of the indicated groups

CCR5^+^CD4^+^Foxp3^GFP^ Tregs injected into Ccr5^−/−^ mice were detected in the CNS and the spleen of recipients, whereas Ccr5^−/−^ recipients receiving CCR5^-^CD4^+^Foxp3^+^ Tregs contained CCR5^−^CD4^+^Foxp3^GFP^ Tregs at a very low frequency (Fig. 8a, b). This result indicates that adoptively transferred CCR5^+^CD4^+^Foxp3^GFP^ Tregs were successfully infiltrated into the lymphoid and inflamed tissues, compared to CCR5^−^CD4^+^Foxp3^GFP^ Tregs. To further characterize CNS inflammation in Ccr5^−/−^ recipients of CCR5^+^ Tregs, the infiltration of CD11b^+^Ly-6C^hi^ monocytes and CD11b^+^Ly-6G^hi^ granulocytes into the CNS was assessed at 5 dpi. Our results revealed that CCR5^+^ Treg-injected Ccr5^−/−^ recipients showed no significant changes in the frequency of CD11b^+^Ly6C^hi^ monocytes and CD11b^+^Ly-6G^hi^ granulocytes during JE progression, compared to those of Ccr5^−/−^ mice receiving CCR5^−^ Tregs (Fig. 8c). Similarly, injection of CCR5^+^CD4^+^Foxp3^GFP^ Tregs into Ccr5^−/−^ recipients resulted in a moderate but insignificant increase in the number of CD11b^+^Ly-6C^hi^ monocytes and CD11b^+^Ly-6G^hi^ granulocytes in the CNS (Fig. 8d). This result indicates that CCR5^+^CD4^+^Foxp3^GFP^ Tregs regulate JE progression in Ccr5^−/−^ mice without affecting CNS infiltration of CD11b^+^Ly-6C^hi^ monocytes and CD11b^+^Ly-6G^hi^ granulocytes. We also enumerated each CD4^+^ Th subset in the CNS. Ccr5^−/−^ recipients of CCR5^+^CD4^+^Foxp3^+^ Tregs had a higher number of CD4^+^Foxp3^+^ Tregs in the CNS at 5 days after infection, compared to Ccr5^−/−^ mice that did not receive Tregs (Fig. 8e). Numbers of IFN-γ^+^CD4^+^ Th1 and IL-17^+^CD4^+^ Th17 cells in the CNS of CCR5^+^ Treg-injected Ccr5^−/−^ mice were not different compared to in the CNS of Ccr5^−/−^ mice, which did not receive Tregs (Fig. 8f). Ccr5^+/+^ mice had an increased number of IFN-γ^+^CD4^+^ Th1 cells, but reduced number of IL-17^+^CD4^+^ Th17 cells compared to Ccr5^−/−^ mice, as shown previously (Fig. 6d). Similarly, an increased number of CD4^+^Foxp3^+^ Tregs was observed in the spleen of CCR5^+^ Treg-injected Ccr5^−/−^ recipients, compared to that of Ccr5^−/−^ mice receiving CCR5^−^ Tregs (Fig. 8g). However, the number of CD4^+^Foxp3^+^ Tregs in Ccr5^−/−^ recipients was lower than that in Ccr5^+/+^ mice. In addition, the injection of CCR5^+^CD4^+^Foxp3^GFP^ Tregs in Ccr5^−/−^ mice did not affect the number of IFN-γ^+^CD4^+^ Th1 and IL-17^+^CD4^+^ Th17 cells in the spleen of Ccr5^−/−^ mice during JE progression (Fig. 8h). Also, a higher proportion of CCR5^+^CD4^+^Foxp3^GFP^ and CCR5^−^CD4^+^Foxp3^GFP^ Tregs adoptively transferred into Ccr5^−/−^ mice were found to produce IL-10, compared to CD4^+^Foxp3^−^ Th cells (Fig. 8i). This indicates that IL-10 production is comparable in CCR5^+^ and CCR5^−^ Tregs purified from Ccr5^+/+^·Foxp3^GFP^ and Ccr5^−/−^·Foxp3^GFP^ mice. Taken together, these results suggest that CCR5^+^CD4^+^Foxp3^+^ Tregs injected to Ccr5^−/−^ mice regulate JE progression by producing IL-10 and enhancing infiltration into lymphoid and CNS tissues, compared to CCR5^−^CD4^+^Foxp3^+^ Tregs.

## Discussion

In this study, we evaluated the role of CCR5 in JE progression. The exacerbation of JE in Ccr5^−/−^ mice was typically associated with a skewed response to IL-17^+^CD4^+^ Th17 cells and correspondingly reduced numbers of CD4^+^Foxp3^+^ Tregs in the spleen and brain. We provided evidence that injection of sorted CCR5^+^CD4^+^Foxp3^+^ Tregs into Ccr5^−/−^ mice ameliorated JE progression without affecting CNS infiltration of IL-17^+^CD4^+^ Th17 cells, myeloid-derived Ly-6C^hi^ monocytes, and Ly-6G^hi^ granulocytes. Instead, adoptive transfer of CCR5^+^CD4^+^Foxp3^+^ Tregs into Ccr5^−/−^ mice increased the expression levels of two anti-inflammatory cytokines, IL-10 and TGF-β, in the spleen and brain. Our results suggest that CCR5 regulates the progression of viral encephalitis via governing a timely and an appropriate CNS infiltration of CD4^+^Foxp3^+^ Tregs and ultimately promoting survival of hosts suffering severe neuroinflammation.

CD4^+^Foxp3^+^ Tregs are believed to maintain host immune homeostasis by actively suppressing pathological and physiological immune responses after homing to inflamed tissues in response to the presence of foreign antigens [21–24]. A putative and somewhat contradictory role of CD4^+^Foxp3^+^ Tregs has been demonstrated in various models of pathogenic infections [23–26]. A putative correlation between CD4^+^Foxp3^+^ Treg levels and the outcome of infectious disease has been reported in WNV encephalitis because patients with symptomatic infection have lower CD4^+^Foxp3^+^ Treg frequencies throughout the infection compared to asymptomatic patients [25]. In addition, a correlation of CD4^+^Foxp3^+^ Tregs with the outcome of flavivirus infection has been reported; Treg expansion but not absolute level was lower in children with severe dengue disease [26]. However, the factors involved in amelioration by CD4^+^Foxp3^+^ Treg of severe flavivirus-induced disease are unclear. Our results suggest a role for CCR5 in regulating JE progression by mediating CD4^+^Foxp3^+^ Treg homing, subsequently inducing skewed IL-17^+^CD4^+^ Th17 responses in lymphoid and inflammatory tissues. Furthermore, considering that asymptomatic and symptomatic populations have similar CD4^+^Foxp3^+^ Treg frequencies prior to WNV infection, while asymptomatic patients exhibit greater Treg expansion within the first 2 weeks of infection [25], the proliferation and/or differentiation of CD4^+^Foxp3^+^ Tregs in asymptomatic persons seems to be promoted by unknown factors (molecular or cellular components) derived from WNV infection. The expanded Tregs then migrate into inflammatory tissues by means of homing receptors such as CCR5, thereby promoting host survival. In line with this notion, the expansion of CD4^+^Foxp3^+^ Tregs in JEV-infected Ccr5^+/+^ mice was around two-fold higher at 5 dpi, and the TLR4 signaling pathway is likely to be involved in their expansion in a JE model [27]. Also, the impact of CCR5 in CD4^+^Foxp3^+^ Treg proliferation and its regulatory role in Treg homing have been clearly demonstrated in a parasitic model [39]. CCR5-dependent recruitment of CD4^+^Foxp3^+^ Tregs may dictate the magnitude of the CD4^+^ Th1 and/or Th17 subset responses to favor a detrimental or beneficial effect on pathogen persistence at the site of infection [23, 24, 40, 41]. Our results favor a beneficial role for CD4^+^Foxp3^+^ Tregs in ameliorating severe neuroinflammation caused by JEV infection, depending on CCR5-mediated homing to inflammatory tissue. Therefore, CCR5 is involved in the putative role of CD4^+^Foxp3^+^ Tregs in severe flavivirus-induced diseases, such as encephalitis and hemorrhagic fever.

The role of CCR5 in infectious diseases is variable in terms of its impact on pathogenesis and disease outcome. An essential role of CCR5 in ameliorating the outcome of infectious diseases has been documented in trypanosomiasis [42], toxoplasmosis [43], genital herpes [44], influenza [45], flaviviral West Nile encephalitis, and JE [16–18], while a beneficial effect of CCR5 deficiency on the outcome of other infectious diseases has been postulated [23, 24, 39, 46, 47]. Mechanistically, these variable outcomes of CCR5 deficiency in infectious diseases have been largely attributed to its regulatory effect on trafficking of leukocytes, including NK, CD4, CD8, and CD4^+^Foxp3^+^ Treg cells to the site of infection as a consequence of the elevated immunopathology. Therefore, this dichotomy in the role of CCR5 in regulating the outcome of infectious diseases prevents the generalization of our findings of the impact of chemokine receptors in disease prognosis. Nevertheless, CCR5 is believed to play a crucial role in protection against severe neuroinflammation caused by flavivirus infections [16–18]. In this study, we also confirmed an essential role for CCR5 in regulating JE progression. However, CCR5 deficiency failed to alter or increase the viral burden in extraneural tissue (spleen) and the CNS. The activation of innate NK cells was increased in Ccr5^−/−^ mice, rather than in Ccr5^+/+^ mice, as corroborated by enumeration of the IFN-γ-producing NK cells. In contrast, Ccr5^+/+^ mice showed a transiently higher number of CNS-infiltrated NK cells with a loss of CD3^−^NK1.1^+^DX5^+^ NK cells in the spleen and blood of both Ccr5^+/+^ and Ccr5^−/−^ mice after JEV infection. Although survived Ccr5^+/+^ mice displayed moderately increased responses of JEV-specific CD4^+^ T cells at 7 dpi, Ccr5^−/−^ mice showed a much higher frequency and number of JEV-specific CD8^+^ T cells in response to stimulation with JEV antigen. These split innate and adaptive immune responses of Ccr5^−/−^ mice during JE progression are contradictory to a previous report that NK cell responses and CD4^+^ as well as CD8^+^ T cell responses decreased in Ccr5^−/−^ mice following JEV infection [17]. The discrepancy might be due to differences in the genetic background and age of the host, strain and dosage of virus, and the route of challenge. Indeed, because Ccr5^+/+^ and Ccr5^−/−^ mice began to show clinical signs, such as neurological disorders, at 3–5 dpi, which is before functional adaptive immune responses were fully induced, the JE model used in this study appeared to have more acute and rapid progression than that in a previous study, in which clinical signs were observed at 8–10 dpi [17]. This accelerated and rapid progression of JE in Ccr5^+/+^ and Ccr5^−/−^ mice might have resulted in induction of distinct NK and CD4/CD8 T cell responses in the host. Early regulation of severe neuroinflammation in the CNS through regulatory mechanisms such as CD4^+^Foxp3^+^ Tregs and myeloid-derived suppressor cells (MDSC) may be important for host survival in cases of acute and rapid progression of JE. This notion is strengthened by the result that Ccr5^+/+^ and Ccr5^−/−^ mice showed similar splenic CD4^+^ and CD8^+^ immune responses in a WNV infection model, to which mice are highly susceptible compared to humans [16]. Also, the fact that CD4^+^Foxp3^+^ Tregs can regulate the progression of WNV encephalitis in an infection model using depletion of CD4^+^Foxp3^+^ Treg cells suggests an important role for CD4^+^Foxp3^+^ Tregs in regulating the progression of fatal neuroinflammation caused by flaviviruses [25]. In this study, the regulatory role of CD4^+^Foxp3^+^ Tregs in JE progression was clarified by adoptive transfer of CCR5^+^CD4^+^Foxp3^+^ Tregs in Ccr5^−/−^ mice. This is strongly supported by a recent report that Tregs can ameliorate encephalitis by repressing effector T cell function [48]. However, the CCR5-mediated regulatory function of CD4^+^Foxp3^+^ Tregs was likely to be relatively unimportant in JE progression, because adoptive transfer of CCR5^+^CD4^+^Foxp3^+^ Tregs between 2 and 4 dpi ameliorated JE progression, whereas CD4^+^Foxp3^+^ Tregs that were adoptively transferred prior to JEV infection rendered the recipients vulnerable to JE (unpublished personal data). Therefore, we used adoptive transfer of CCR5^+^CD4^+^Foxp3^+^ Tregs in Ccr5^−/−^ mice at 3 dpi, the time point at which infected mice began to show clinical signs, such as generalized piloerection, paresis, and rigidity. Although further study is warranted, CCR5 appears to play a non-committed role in JE progression by regulating the trafficking equilibrium of effector leukocytes and regulatory CD4^+^Foxp3^+^ Tregs, depending on disease progression.

It is likely that our results discount the role of CCR5 in ameliorating JE progression by CNS infiltration of effector leukocytes such as NK cells, macrophages, CD4^+^ cells, and CD8^+^ T cells. It has long been assumed that leukocyte infiltration into the CNS is critical for clearing virus and aiding recovery. CCR5 deficiency was associated with increased flavivirus burden in the CNS but not in extraneural tissues, which was mechanistically mediated by inappropriate CNS infiltration of leukocytes [16, 17]. However, the critical role of CCR5 in flavivirus pathogenesis appears to be unique in other neurotropic viruses, because infections of Ccr5^−/−^ mice with several neurotropic viruses, such as LCMV [49], retrovirus FR98 [50], and mouse hepatitis virus (MHV) [51], resulted in viral burdens in the CNS similar to those of Ccr5^+/+^ mice. Unlike earlier works on flavivirus encephalitis [16, 17], the present study showed that the JEV burden in the extraneural tissue and CNS of Ccr5^+/+^ mice was similar to that in Ccr5^−/−^ mice, with transiently and early increased CNS infiltration of NK and CD4^+^ cells, but not CD8^+^ T cells, in Ccr5^+/+^ mice. Although the mechanisms of increased CNS infiltration of leukocytes in Ccr5^−/−^ mice with an unchanged viral burden need to be defined, the JE model used appears to affect the dynamics of leukocyte CNS infiltration and the subsequent viral burden. CNS trafficking of Ly-6C^hi^ monocytes and Ly-6G^hi^ granulocytes is mediated through a multistep process governed by CC and CXC chemokines. In support, the enhanced expression of CC chemokines including CCL2, CCL3, CCL4, and CCL5 appeared to facilitate CNS infiltration of Ly-6C^hi^ monocytes in Ccr5^−/−^ mice at the early phase, although whether these cells function to suppress or promote pathogenesis is unclear [52, 53]. One interesting result in this study was the reversal of CXC chemokine expression between 3 and 5 dpi. Although CXCL1 and CXCL2 play a dominant role in the trafficking of Ly-6G^hi^ granulocytes, CC chemokines are also likely to be involved in CNS infiltration of Ly-6G^hi^ granulocytes [54]. Furthermore, increased CNS infiltration of Ly-6G^hi^ granulocytes in Ccr5^−/−^ mice is strengthened by the result that CCR5 ablation increases the recruitment of Ly-6G^hi^ granulocytes in herpetic encephalitis [55]. Also, it is conceivable that CXCL2 is involved in the recruitment of granulocytic MDSCs at a later stage [56], thereby resulting in the amelioration of JE progression. However, CCR5 appeared not to be involved in the migration of myeloid and lymphoid cells from the blood into the brain, because the accumulation of myeloid (monocytes, granulocytes) and lymphoid (NK, CD4/CD8 T cells) cells in the blood showed similar patterns to those in the brain. These data are in line with a previous report that CCR2 is not involved in monocyte migration from the blood into the brain [57]. CCR5 ablation may cause accumulation of CCR5 ligands (CCL3, CCL4, CCL5) via their compensation mechanism [58], which could induce dysregulation of the migration of monocytes, NK cells, and T cells expressing cognate receptors (CCR1, CCR3). Also, the appropriate adaptive CD4^+^ and CD8^+^ T cell responses can be achieved by orchestrated chemokine expression in secondary lymphoid tissues to promote contact between T and dendritic cells [59]. Indeed, our results demonstrate unexpected adaptive JEV-specific CD4^+^ and CD8^+^ T cell responses in Ccr5^−/−^ mice; stronger responses of JEV-specific CD4^+^ T cells were induced in Ccr5^+/+^ mice, whereas Ccr5^−/−^ mice displayed a potent JEV-specific CD8^+^ T cell response. Ultimately, these speculations suggest that other chemokine receptors may be involved in the migration of myeloid and lymphoid cells as well as adaptive T cell responses in a CCR5-ablated environment, due to redundancy and compensation of chemokines and their receptors. In addition, the role of NK and CD8^+^ T cells in CNS clearance of JEV remains elusive, because the depletion or adoptive transfer of NK and CD8^+^ T cells does not contribute significantly to host survival or viral clearance [36]. Also, although IFN-γ^+^CD4^+^ Th1 cells are believed to play a role in regulating JE progression by reducing viral burden in the CNS [38], only co-transfer of immune CD4^+^ and CD8^+^ T cells, not individual transfer of either T cell subpopulation, significantly ameliorates JE progression and promotes host survival [37]. These facts support the possibility that transiently and early increased CNS infiltration of NK and IFN-γ^+^CD4^+^ Th1 cells in Ccr5^+/+^ mice may not be sufficient for viral clearance from the CNS, resulting in a similar CNS viral burden in Ccr5^+/+^ and Ccr5^−/−^ mice. Therefore, balanced and orchestrated CNS infiltration by innate NK and adaptive T cell subpopulations likely mediates viral clearance, thereby providing protection against JE progression without tissue injury.

IL-17 is produced mainly by IL-17^+^CD4^+^ Th17 cells. It plays a critical role in autoimmune and virus-caused immunopathologic diseases by facilitating neutrophil recruitment [60–62]. In contrast, IL-17 appears to play a minor role in protective immunity against parasitic infection [63] but a more important role in fungal infection in a CCR5-ablated environment [39]. In the present study, CD4^+^Foxp3^+^ Tregs did not directly regulate CNS recruitment of IL-17^+^CD4^+^ Th17 cells or their IL-17 production. In addition, adoptive transfer of CCR5^+^CD4^+^Foxp3^+^ Tregs did not influence CNS infiltration of Ly-6C^hi^ monocytes and Ly-6G^hi^ granulocytes. However, the anti-inflammatory cytokines IL-10 and TGF-β produced by adoptively transferred CCR5^+^CD4^+^Foxp3^+^ Tregs might have played a role in regulating JE progression. This notion is supported by the finding that insufficient anti-inflammatory cytokine levels are associated with exacerbated JE [64]. Furthermore, our data are strongly supported by the finding that IL-10 ablation exacerbates alphavirus encephalomyelitis by enhancing CNS infiltration of IL-17^+^CD4^+^ Th17 and IFN-γ^+^CD4^+^ Th1 cells, without affecting the amount of brain inflammation and viral replication [65]. Indeed, the majority of adoptively transferred CCR5^+^CD4^+^Foxp3^+^ Tregs in Ccr5^−/−^ mice produced IL-10. Furthermore, generation of CD4^+^Foxp3^+^ Tregs and IL-17^+^CD4^+^ Th17 cells is reciprocally regulated [60–62]. This developmental link between CD4^+^Foxp3^+^ Tregs and IL-17^+^CD4^+^ Th17 cells has led to speculation that these T cell subsets exist in equilibrium during inflammation and infection [66, 67]. However, this equilibrium was disturbed, thereby causing exacerbation of JE progression in Ccr5^−/−^ mice. Therefore, our results provide insight into the utility of IL-10 and CD4^+^Foxp3^+^ Tregs for regulating JE progression by maintaining the balance between CD4^+^Foxp3^+^ Tregs and IL-17^+^CD4^+^ Th17 cells at specific time points.

## Conclusions

CCR5 could regulate JE progression via mediating the equilibrium between CD4^+^Foxp3^+^ Tregs and IL-17^+^CD4^+^ Th17 cells without causing tissue injury. This critical and extended role of CCR5 in flavivirus-induced diseases raises possible safety concerns regarding the use of CCR5 antagonists in HIV individuals who inhabit regions where both HIV and flaviviruses such as JEV and WNV are endemic [68]. Therefore, understanding the detailed role of CCR5 in the pathogenesis of flavivirus-caused encephalitis will be important to prevent any potential risk to HIV-positive individuals who take CCR5 antagonists with curative intent.

## Abbreviations

BBB, blood–brain barrier; CCR5, CC chemokine receptor 5; CNS, central nervous system; dpi, days post-infection; HIV, human immunodeficiency virus; JEV, Japanese encephalitis virus; KO, knockout; LCMV, lymphocytic choriomeningitis virus; MDSC, myeloid-derived suppressor cell; Treg, regulatory T cell; WNV, West Nile virus
